# RETRACTION: Excess mortality across countries in the Western World since the COVID-19 pandemic: ‘Our World in Data’ estimates of January 2020 to December 2022

**DOI:** 10.1136/bmjph-2023-000282ret

**Published:** 2026-08-11

This article^1^ is retracted by the journal.

## What happened after publication

The journal was alerted to concerns about quality, originality and messaging in this research paper. The journal published an expression of concern^2^ to explain that the research does not investigate or establish a causal link between COVID-19 vaccination and mortality. The authors’ institution issued a press release on 11 June 2024 confirming they would investigate the work.

### Institutional investigation

The Princess Máxima Centre was the institution of three of the four authors and their investigation was based on the Netherlands Code of Conduct for Research Integrity.^3^ The institution sent the anonymised report describing their investigation into the study’s quality and the conduct of two of the authors Gertjan Kaspers and Minke Huibers to BMJ in May 2025. The report says that the institution did not investigate Marcel Hoogland because he was not affiliated with the Princess Maxima Center or Saskia Mostert because she was not available to co-operate with the institutional investigation. The report is available as a supplementary file to this retraction notice. The institution asked BMJ to return or destroy the full report of their investigation, which had been sent to the journal outside of the agreed procedure at the end of 2024.

10.1136/bmjph-2023-000282ret.supp1 Supplementary data

The anonymised report does not comment on the quality or originality of the work in detail. However, the report concluded that there was no evidence of malicious intent, fabrication or falsification of data, or plagiarism on the part of the individuals who were investigated. Regarding the messaging, the institution concluded in the report that the article pays disproportionate attention to the possibility that COVID-19 containment measures and vaccination might have caused excess mortality; the author's work did not investigate the causes for excess mortality. The article misleadingly cited literature that supported its own arguments, but did not sufficiently discuss relevant literature that supported alternative interpretations.The institution, as well as Kaspers and Hubers, requested retraction due to concerns about misinterpretation of the paper by others and subsequent public health risk. This is not in keeping with COPE guidance for retraction. In June 2026, while communicating with the institution about the retraction, the institution made comments about Mostert’s conduct which were not included in the report. They told BMJ that from the available evidence, the committee concluded that Mostert’s conduct qualified as questionable behaviour and that she gave disproportionate attention to the possibility that vaccination might have caused excess mortality. The institution did not respond to follow up emails from BMJ for clarification given prior statements to BMJ and the official report stated that Mostert was not investigated by the institution.

### Journal investigation

Saskia Mostert was the corresponding author and guarantor of the work, therefore, the journal wanted to understand her perspective on key issues. In July 2024 her co-authors informed BMJ that she was unavailable. The report from the institution shared in May 2025 stated the same. BMJ wrote to her again in October 2025 and she replied.Regarding the originality of the work, Mostert said much of the work was conducted by others, however, her opinion was that this was clear from the time of submission. The data were prepared by ‘Our World in Data’, Human Mortality Database and World Mortality Database using the estimate model of Karlinski and Kobak. The journal concluded that the extent of reuse was not made sufficiently clear, and that the amount of original research conducted by the authors was limited.The journal reviewed the messaging around the causes of excess mortality in the paper. Following submission of the work by the authors, and its internal and external review at the journal, Mostert revised the discussion of causes of excess mortality in response to reviewer comments. Mostert added text and references to the work which was resubmitted and later accepted for publication. Discussing the paper with the journal post-publication, Mostert suggested to the journal that, from her perspective, by adding studies “indexed on PubMed”, published by “investigators from respectable institutes” in international, peer reviewed journals, the references did not include misinformation. Although Mostert stands by her work, she was willing to modify text connected to containment measures and vaccines to make it more balanced. However, the journal’s view is that this would be insufficient.Overall, the journal concludes that the discussion of causes of mortality is imbalanced, lacks rigour, and includes misinformation.

## Reasons for retraction

The article is retracted by the journal for misinformation in the discussion regarding the possible causes of excess mortality, and because the limited nature of the original work by the authors was not sufficiently described.

## Further action and comments

The authors’ institutions have been notified of the retraction.

Kaspers and Huibers agree with the retraction. However, they do not agree that the article contains misinformation. Writing on behalf of Hoogland and herself, Mostert said that she disagrees with the decision to retract and the reasons for retraction.

The time to investigate this paper was lengthy because the journal first awaited the formal official outcome of the institutional report. This report did not comment on Mostert, who was the corresponding author and guarantor of the work. When the report became available, it was fair to contact Mostert’s again for her perspective on key issues before reaching a final verdict.

## References

[1] Mostert S, Hoogland M, Huibers M, et al. Excess mortality across countries in the Western World since the COVID-19 pandemic: ‘Our World in Data’ estimates of January 2020 to December 2022. BMJ Public Health 2024;2:e000282. 10.1136/bmjph-2023-000282 PMC13482250 42614536

[2] Expression of concern: Excess mortality across countries in the western world since the COVID-19 pandemic: ‘Our World in Data’ estimates of January 2020 to December 2022. BMJ Public Health 2024;2:e000282eoc. 10.1136/bmjph-2023-000282eoc PMC13482250 42614536

[3] KNAW, NFU, NWO, *et al*. Netherlands code of conduct for research integrity. 2018. Available: https://www.nwo.nl/en/netherlands-code-of-conduct-for-research-integrity

