# Supplementary material for: RETRACTION: Excess mortality across countries in the Western World since the COVID-19 pandemic: ‘Our World in Data’ estimates of January 2020 to December 2022
Source: BMJ Public Health. 2026 Aug 11;4(3):e000282ret. doi: 10.1136/bmjph-2023-000282ret (PMC13479481; doi:10.1136/bmjph-2023-000282ret)
Supplement: Supplementary file 1 [file bmjph-4-3-s001.pdf]

Anonymized Report
Committee for Scientific Integrity Princess Máxima Center
2025/01/31

Case description
In June 2024, the Scientific Integrity Committee of the Princess Máxima Center (hereafter: CWI) received a complaint from an external complainant (hereafter: Complaint) and a request for investigation (hereafter: Request) from the Board of Directors regarding a possible violation of integrity by three researchers of the Princess Máxima Center (hereafter: Defendants). Various concerns were raised regarding a manuscript entitled 'Excess mortality across countries in the Western World since the COVID-19 pandemic: 'Our world in data' estimates of January 2020 to December 2022.' Published in the British Medical Journal (BMJ).

Procedure followed
The CWI declared the complaints admissible and launched an investigation. The CWI requested information from the defendants and internal departments of the Máxima. One of the three defendants was not available to cooperate with the investigation, after which it was decided to postpone the investigation of this defendant and continue the investigation of the other two defendants. The CWI sought the advice of an independent, external expert, who prepared a report based on the information submitted. The CWI shared the information from the internal departments and the expert report with those involved and provided the opportunity for a written defense. Following this, the CWI held hearings with the defendants and the complainant.
Based on the information received, the expert report and the defendants' written and oral defenses, the CWI prepared an advisory report and shared this with the Board of Directors. The Board adopted the advice and shared the initial judgement with those involved. Those involved waived the option of seeking the opinion of the National Organization for Scientific Integrity (LOWI), after which the conclusions of this report were declared final.

Summary Request/Complaint
The cause that led to the filing both the Request and the Complaint was the publication of manuscript describing the extent of excess mortality since the Covid-19 pandemic in a peer-reviewed scientific journal. Several of the authors of this article were (partly) affiliated with the Princess Máxima Center at the time of publication. The article caused much commotion because it may suggest that there is a link between excess mortality and vaccinations.

Summarized Outcome of Investigation
The CWI investigated the case using the guiding principles laid out in the Dutch Code of Conduct for Scientific Integrity (hereafter: Code of Conduct). In doing so, the CWI requested an external expert to provide an independent opinion on the case and to draw up a report based on her findings. Based on the expert report, the CWI concludes the following:

BMJPH 2026; 4:e000282ret. doi: 10.1136/bmjph-2023-000282ret

Princess máxima center pediatric oncology

- The study analyzes the level of excess mortality in the 2020-2023 period in the Western World, but no research was conducted to actively investigate the possible causes of excess mortality. It is not an explanatory study, but merely a quantification of the excess mortality. That excess mortality occurred, however, was known and not newsworthy. Despite the fact that the authors do not investigate the causes for excess mortality, the introduction and discussion of the article are largely focused on how to explain the excess mortality. In discussing possible causes, the article pays disproportionate attention to the possibility that introduction of corona measures and vaccination might have caused excess mortality, whilst largely ignoring alternative explanations.
- The article misleadingly and unilaterally cites literature that supports its own arguments. Important, relevant literature that supports alternative views, interpretations and theories is not discussed, or is discussed far too little.

Based on the expert report, hearings and further research, the CWI has found that 14 of the 21 standards explicitly examined were violated. However, no evidence was found for malicious intent, fabrication or falsification of data, or plagiarism.

Conclusion

The CWI concludes that the authors' actions do not qualify as scientific misconduct, since no evidence was found of malicious intent, fabrication or falsification of data, or plagiarism. However, 14 of the 21 standards of the Code of Conduct were violated, and the committee therefore qualified the actions of one of the authors as questionable behavior, and the actions of another author as a shortcoming. This conclusion was reached using the weighing criteria as explained in Chapter 5 of the Code of Conduct. Factors that weighed in the judgement were the high degree of non-compliance with the code of conduct; the negative effect on confidence in science; the negative impact on society and the concern of a serious scientific publication in a reputable journal. In contrast, for both defendants there was no malicious intent, but the result was due to carelessness, ignorance and negligence. The different responsibilities the authors carried for the published work led to a difference in judgement.

BMJPH 2026; 4:e000282ret. doi: 10.1136/bmjph-2023-000282ret
